# Facilitating effects of plant extracts on soil health and replanted *Panax ginseng* growth in recession soil

**DOI:** 10.1371/journal.pone.0311679

**Published:** 2024-10-07

**Authors:** Ergang Wang, Yi Zhou, Xinyue Miao, Guixiang He, Pengyuan Lv, Lixiang Wang, Yu Zhan, Changbao Chen, Qiong Li

**Affiliations:** 1 Jilin Ginseng Academy, Changchun University of Chinese Medicine, Changchun, China; 2 School of Pharmaceutical Sciences, Changchun University of Chinese Medicine, Changchun, China; Portuguese Catholic University: Universidade Catolica Portuguesa, PORTUGAL

## Abstract

**Background:**

Plant extracts have been shown to be effective agricultural strategies for improving soil fertility and quality, and promoting plant growth in soil degradation remediation. The application of plant extracts improves the material cycle of soil microecology, such as the decomposition of nitrogen, phosphorus, and potassium, while increasing plant resistance. However, there is currently no experiment to demonstrate whether plant extracts have a promoting effect on the growth of ginseng and the mechanism of action.

**Objectives and methods:**

Pot experiments were carried out to investigate the effects of extracts, namely *Rubia cordifolia* (RC), *Schisandra chinensis* (SC), and *Euphorbia humifusa* (EH) on soil properties, enzyme activities, and plant physiological characteristics were evaluated.

**Results:**

Results showed that compared with CK, plant extract-related treatments increased soil Organic carbon (OC), Available nitrogen (AN), Available phosphorus (AP) contents, and Soil urease activity. (S-UE), Soil sucrase activity (Soil sucrase), Soil acid phosphatase activity. (S-ACP). Meanwhile, plant extract-related treatments significantly increased plant physiological properties and TP (Total protein) content, and decreased the content of MDA (malondialdehyde) by 15.70% -36.59% and PRO (proline) by 30.13% -148.44%. Furthermore, plant extract-related treatments also significantly promote plant growth and reduce plant incidence, the fresh weight of ginseng increased by 27.80% -52.08%, ginseng root activity increased by 45.13% -90.07%, and ginseng incidence rate decreased by 20.00% -46.67%. Through correlation analysis between fresh weight of ginseng and root parameters and soil index, fresh weight is significantly positively correlated with root diameter, fiber root number, root activity, total protein (TP), catalytic activity (CAT) and superoxide dismutase activity (SOD), H, soil urea activity (S-UE), soil sucrose activity (S-SC), soil acid phosphate activity (S-ACP), and soil laccase activity (SL); The fresh weight was significantly negatively correlated with incidence rate, disease severity index, and malondialdehyde content (MDA).

**Conclusion:**

In summary, plant extract-related treatments improve soil quality and promote ginseng growth, further enhancing soil health and plant disease resistance. These findings provide new insights into ginseng cultivation and soil health management and highlight a new approach that can be applied to a wider range of agricultural practices and environmental sustainability.

## Introduction

Ginseng (*Panax ginseng* C. A. Mey.) is a perennial herb with broad application prospects in ensuring people’s health and life safety [1]. Due to increased demand and inadequate wild resources, it has been intensively cultivated in China, Russia, North Korea, South Korea, and other places [2]. Unfortunately, continuous monoculture and over-fertilization have resulted in soil degradation and accumulation of soil-borne pathogens [3–5], which jeopardizes ginseng production [6]. Therefore, effective improvement measures are crucial for maintaining the productivity of ginseng under intensive cultivation.

Soil chemical fumigation technology is currently the most widely used method in production to suppress soil-borne pathogens. Chloropicrin is one of the most commonly used soil fumigants. Studies have shown that chloropicrin fumigants can kill more than 85% of bacteria, fungi, and actinomycetes in soil [7], and also control weeds and nematodes [8]. However, it has no selectivity and can kill pathogenic bacteria in the soil, but also beneficial bacteria, which can easily endanger human health [9]. With increasing attention to sustainable agricultural development and human health, traditional chemical fumigants have been gradually phased out. Therefore, we urgently need a healthy soil management measure to replace reliance on chemical fumigants. Plant extracts are often considered a promising soil health management practice that extracts active ingredients from plant roots, stems, leaves, flowers, and other parts to kill soil-borne pathogenic microorganisms, improve soil physicochemical properties, and promote plant growth [10–13]. For example, plant extracts improved the biochemical index of corn, reduced the impact of abiotic stress, and promoted the establishment and growth of corn seedlings [14]. After the application of plant extracts, the enhanced nutritional growth, chlorophyll concentration, aboveground biomass, and yield properties of potatoes can also be attributed to their biological stimulating effects, which improves the physiological resistance of plants and the physicochemical properties of soil [15]. Seaweed extract application improved the activities of antioxidant enzymes, including SOD, CAT, and POD, in roots and alleviated the oxidative damage of roots caused by drought stress, seaweed extract application can improve soil physical structure, promote enzyme activities, and increase the available N, P, and K of the soil, promote the growth of sugarcane [16].

Soil is the foundation for the growth of perennial plants such as ginseng and an important source of plant nutrition [17]. Studies has shown that a deteriorating soil environment can lead to inhibition of plant photosynthesis [18], imbalance of reactive oxygen species metabolism [19], damage to antioxidant enzyme systems [20], and imbalance of hormone levels [21]. Meanwhile, plants continuously absorb soil nutrients during the continuous monoculture process, causing a continuous decrease in soil nutrients in the root zone [22], leading to soil nutrient imbalance and element proportion imbalance [23]. Soil enzymes are closely related to soil nutrients, which together constitute the soil microecological environment and affect plant root activity [24, 25]. Additionally, with the application of a large number of pesticides and fertilizers, soil salt continuously accumulates towards the surface of the soil [26], accelerating soil acidification [27], leading to severe soil salinization and hardening [28], ultimately leading to poor crop development. Thus, it is particularly imperative to explore the effects of different plant extracts on soil enzyme activity, soil nutrients, and ginseng growth and development.

We speculate that: (1) Different plant extracts have different effects on soil properties and the growth of ginseng; (2) Plant extracts can improve soil health; (3) Plant extracts enhance ginseng root activity, promote the growth of ginseng, and reduce the incidence rate of ginseng. To test these hypotheses, we conducted field investigations and selected three companion plants(*Rubia cordifolia*, *Schisandra chinensis*, and *Euphorbia humifusa*) of ginseng for experiments and we selected a soil where ginseng seedlings had a survival rate of less than 30% after six years of continuous cultivation and studied the effects of extracts on soil physicochemical properties, enzyme activity, and seedling growth after replanting.

## Materials and methods

The experiments involved in this study were carried out at the Jilin Ginseng Academy of Changchun University of Chinese Medicine, and the work permits of relevant institutions were not involved. It is hereby declared that the situation is true.

### Experimental design

The dark brown forest soil used in this study was collected from Baixi Forest Farm (44˚05ʹN, 127˚67ʹE) in Fusong County, Baishan City, Jilin Province, China. The area belongs to a temperate continental monsoon climate. The annual average temperature is 5°C, the highest temperature is 34°C, the lowest temperature is -36°C, the annual average precipitation is 712 mm, the frost-free period is 79–150 days, and the soil parent material type is dark brown soil. The soil has been continuously planted with ginseng for six years and suffered severe disease, and soil samples have been collected after harvest. Soil’s properties were as follows: pH 4.8; electrical conductivity (EC), 157.8 μS/cm; organic carbon (OC), 12.30 g/kg; and available phosphorus (AP), 2.73 mg/kg.

Prior to the pot experiment, the *Rubia cordifolia*, *Schisandra chinensis*, and *Euphorbia humifusa* were crushed (particle size < 2 mm). Plant powder was separated accurately weighed 10.0 g and placed in a beaker, and then 100 mL of water was added to extract in a 60°C constant temperature water bath for 2 hours. After filtering, the residue was extracted twice with 100 mL of water, combined with three filters, concentrated to 100 mL, obtained 0.1g/mL of extractions, stored at 4°C for later use.

The pot experiment was conducted in the artificial climate chamber of Changchun University of Traditional Chinese Medicine on December 26, 2022. Pots (17 cm × 17 cm × 14 cm, without drainage holes) were filled with 1.8 kg soil, and two-year-old healthy ginseng seedlings of similar size were transplanted, with 3 plants per pot. Four treatments: (1) CK, untreated soil; (2) RC, soil with 30 mL *Rubia cordifolia* extract, (3) SC, soil with 30 mL *Schisandra chinensis* extract, (4) EH, soil with 30 mL *Euphorbia humifusa* extract. There were three replications of these treatments, which were established randomly. The *Rubia cordifolia*, *Schisandra chinensis*, and *Euphorbia humifusa* were purchased from Beijing Tong Ren Tang Traditional Chinese Medicine Co., Ltd (Beijing, China). Dilute 10 mL of the three plant extracts with water every 30 days to 1L, and then add them to the potted soil until the ginseng is harvested. The total amount of each extract is 30 ml.

### Sample collection and processing

Soil samples and plants from four treatments were collected during the harvesting period (March 26, 2023), and three replicates of each treatment were mixed as composite samples. Sample the soil around the roots of ginseng using a five-point method. The sampling depth was 5–8 cm. Collect 3 times for each treatment and homogenize the collected soil to obtain a composite sample. Uniformly collected samples are naturally dried and sieved through a 2 mm sieve to measure soil physicochemical properties and enzyme activity. Meanwhile, separate the aboveground and underground parts of the plant, wash gently, and dry the roots with absorbent paper. Subsamples were stored at -80°C for physiological and biochemical analysis.

### Soil physicochemical properties analysis

Soil pH and electric conductivity (EC) were measured by a pH meter (PHSJ-3F, Shanghai, China) and conductivity meter (DDS-307A, Shanghai, China) at a soil-water ratio of 1:5 (weight/volume), respectively. Soil organic carbon (OC) was determined by the potassium dichromate external heating method [29]. Soil available nitrogen (AN) was determined by the alkaline diffusion method [30]. Soil available phosphorus (AP) was determined by the NaHCO_3_ extraction molybdenum antimony colorimetry method [31].

### Soil enzyme activity analysis

Soil urease activity (S-UE) was determined by indophenol blue colorimetry, and the activity was defined as 1μg NH_3_-N produced per gram of soil per day [32]. Soil sucrase activity (S-SC) was determined by 3,5-dinitro salicylic acid colorimetry, and the activity was defined as 1 mg of reducing sugar produced per gram of soil per day at 37°C [33]. Soil acid phosphatase activity (S-ACP) was measured by disodium phenyl phosphate colorimetry, and the activity was defined as 1 nmol phenol release per gram of soil per day at 37°C as one enzyme activity [34]. Soil laccase activity (SL) was measured by activity spectrophotometry, and defined as the amount of enzyme required to oxidize 1 nmol of substrate ABTS per minute per gram of soil [35].

### Plant growth and incidence survey

During the harvesting period, ginseng growth conditions below ground were recorded. The measurement and recording methods are as follows: On the day of sampling, the main root length of ginseng was measured with a straightedge, the diameter of the root was measured with a vernier caliper, and weighed the fresh weight of a single ginseng using a balance, while the number of fibrous roots was measured. Meanwhile, five ginseng plants were randomly selected from each treatment to investigate the incidence of root disease in the underground part of ginseng, and the incidence area of each root was graded. A indicates no visible root lesions, B indicates root lesions 0.9 mm in diameter (brown), C indicates root lesions 1–4.0 mm (dark brown), D indicates root lesions 4–7.0 mm (black), E indicates interfusion of root lesions, and F indicates root complete decay [36]. The calculation equation is as follows:

$$\mathrm{Disease}\;\mathrm{severity}=\frac{\left(\mathrm{Xa}\times 1)+(\mathrm{Xb}\times 2)+(\mathrm{Xc}\times 3)+(\mathrm{Xd}\times 4)+(\mathrm{Xe}\times 5)+(\mathrm{Xf}\times 6\right)}{\mathrm{Xa}+\mathrm{Xb}+\mathrm{Xc}+\mathrm{Xd}+\mathrm{Xe}+\mathrm{Xf}}$$

$$\mathrm{Incidence}\;\mathrm{rate}=\frac{\mathrm{disease}\;\mathrm{plants}}{\mathrm{Total}\;\mathrm{number}\;\mathrm{of}\;\mathrm{plants}\;\mathrm{surveyed}}\times 100\%$$

Where Xa, Xb, Xc, Xd, Xe, and Xf represent the numbers of plants with rotting severity of a, b, c, d, e, and f, respectively.

### Root activity analysis

The fresh plant root sample was used to assess root activity by the 2,3,5-triphenyl tetrazolium chloride (TTC) method [37]. TTC’s oxidation state is colorless by itself. We soaked the roots in TTC aqueous solution, and TTC entered the root cells. This test is based on dehydrogenase in live roots reducing colourless TTC to red triphenyl formazan. Afterward, the compound is extracted by a spectrophotometer at 485 nm after a fixed incubation period.

### Plant physiological properties analysis

Total protein content (TP), catalase activity (CAT), superoxide dismutase activity (SOD), peroxidase activity (POD), malondialdehyde content (MDA), and proline (PRO) were used to indicate the physiological properties of the plant. The activities of plant SOD, POD, CAT, and the content of TP, MDA, and PRO were measured using a kit produced by the company Nanjing Jiancheng Bioengineering Institute (Nanjing, China). TP is defined as the protein content per g of tissue. POD activity units are defined as the amount of enzyme that catalyzes 1 μg of substrate per minute per g of tissue at 37°C. CAT activity units are defined as the breakdown of 1 μmol of H_2_O_2_ per second per g of tissue. SOD activity units are defined as the corresponding amount of SOD per g of tissue when the SOD inhibition rate reaches 50% in 1 mL of reaction solution. MDA is defined as the content of malondialdehyde per g of tissue. PRO is defined as the proline content per g of tissue.

### Data analysis

Using IBM SPSS 21.0 statistical software (SPSS Inc, USA), we measured differences in soil physicochemical properties, soil enzyme activity, and ginseng physiological characteristics between different treatments using one-way analysis of variance (ANOVA) (*P* < 0.05). GraphPad Prism (Version 8.01) is used to create all graphics. Correlation heatmap created using Majorbio platform.

## Results

### Physicochemical properties of the rhizosphere soils

Analysis of the collected soil samples showed a clear difference in physicochemical properties changes after plant extract-related treatments of ginseng planted soil (*P* < 0.05, Table 1). Soil pH was significantly higher in the RC treatment than in the CK treatment (*P* < 0.05), but the difference in soil pH among the SC, EH treatment, and CK treatment was not significant (Table 1). Soil EC was significantly lower in the RC and EH treatment than in the CK treatment (*P* < 0.05), but the difference between the SC and CK treatment was not significant, with the RC treatment having the lowest soil EC (Table 1). Soil OC content was significantly increased in all treatments compared to the CK treatment (*P* < 0.05), and the EH treatment had the highest soil OC value (Table 1). Soil AN content increased significantly (*P* < 0.05) in all treatments compared to the CK treatment, and the EH treatment had the highest soil AN content (Table 1). Compared with the CK treatment, soil AP content was significantly increased in the RC, SC, and EH treatment (*P* < 0.05), and the highest AP content was found in the SC treatment (Table 1).

**Table 1:** Physicochemical properties of rhizosphere soils under different treatments.

Treatment	pH	EC (μS/cm)	OC (g/kg)	AN (mg/kg)	AP (mg/kg)
CK	4.93±0.03^b^	166.00±3.78^a^	7.98±0.81^c^	195.25±17.22^c^	4.31±0.15^d^
RC	5.40±0.10^a^	114.20±2.21^c^	13.46±0.81^b^	286.13±8.71^b^	5.41±0.27^c^
SC	4.95±0.02^b^	163.85±1.36^a^	13.13±1.70^b^	285.25±8.75^b^	7.38±0.16^a^
EH	4.95±0.02^b^	154.08±2.48^b^	16.78±1.69^a^	334.25±3.91^a^	6.49±0.30^b^

Values (mean ± SD, n = 3) within the same column followed by different letters are significantly different at *P* < 0.05 according to the one-way ANOVA. EC, electric conductivity; OC, organic carbon; AN, available nitrogen; AP, available phosphorus. CK, untreated soil; RC, *Rubia cordifolia* extract; SC, *Schisandra chinensis* extract; EH, *Euphorbia humifusa* extract

### Enzyme activity of the rhizosphere soils

**Fig 1:**
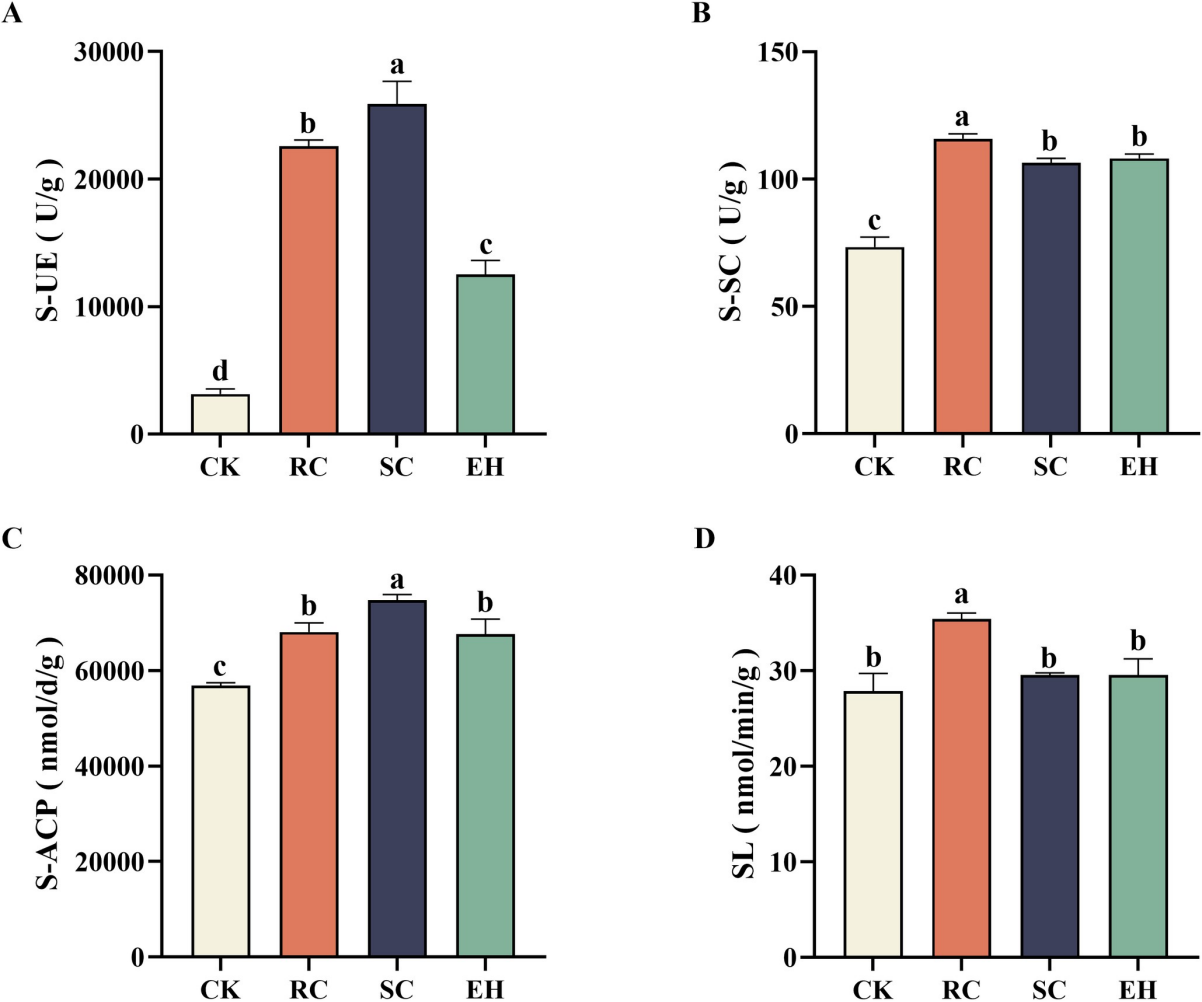
Enzyme activity of rhizosphere soils under different treatments. The error bars indicate the standard errors of the means of three replicates. The one-way ANOVA indicates that the letters denote a significant difference at *P* < 0.05. S-UE, soil urease; S-SC, soil sucrase; S-ACP, soil acid phosphatase; SL, soil laccase. CK, untreated soil; RC, *Rubia cordifolia* extract; SC, *Schisandra chinensis* extract; EH, *Euphorbia humifusa* extract.

Analysis of the collected soil samples showed a clear difference in enzyme activity changes after plant extract-related treatments of ginseng planted soil (*P* < 0.05, Fig 1). S-UE activity was significantly increased in RC, SC, and EH treatment compared to the CK treatment (*P* < 0.05), with the highest soil S-UE activity in the SC treatment (Fig 1A). S-SC activity increased significantly (*P* < 0.05) in all treatments compared to the CK treatment, with the highest S-SC activity in the RC treatment and the lowest S-SC activity in the SC treatment (Fig 1B). S-ACP activity was significantly increased in all treatments compared to the CK treatment (*P* < 0.05) (Fig 1C). Compared to the CK treatment, SL activity was significantly increased in the RC treatment (*P* < 0.05), and slightly increased in SC and EH treatment, but the difference was not significant (Fig 1D).

### Growth and incidence rate of ginseng

**Fig 2:**
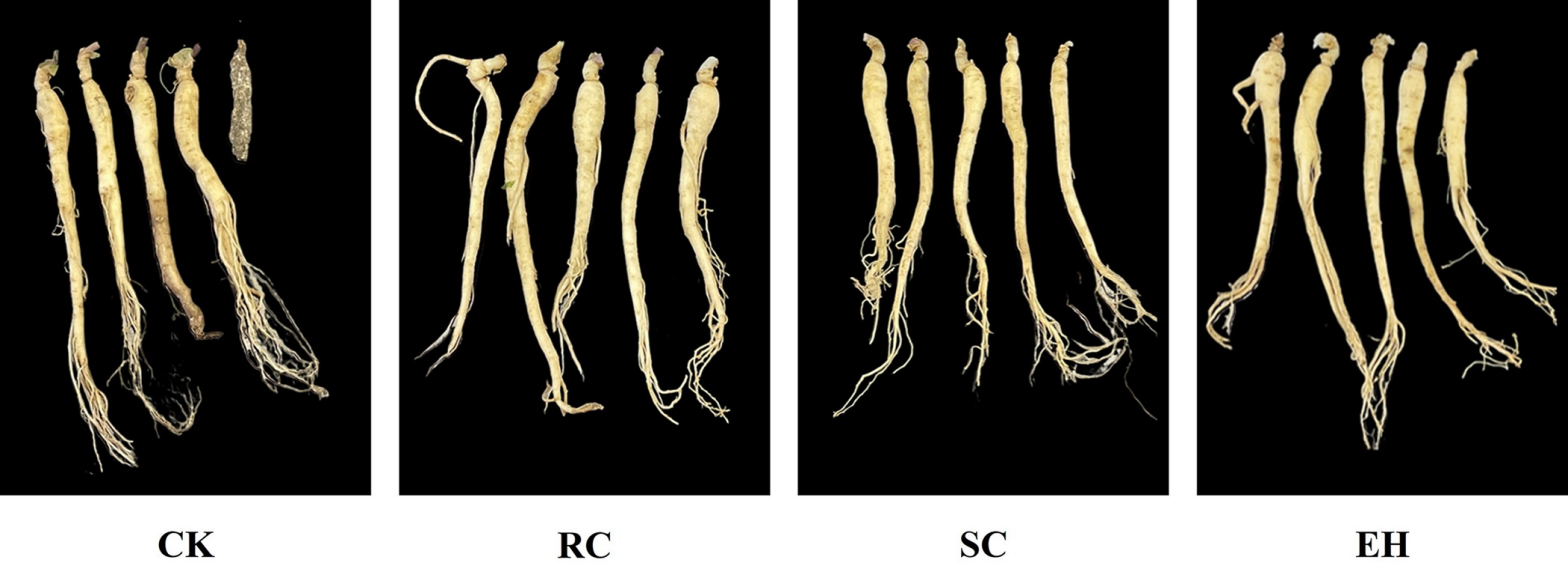
Growth of ginseng under different treatments during harvest. The error bars indicate the standard errors of the means of three replicates. The one-way ANOVA indicates that the letters denote a significant difference at P < 0.05. CK, untreated soil; RC, *Rubia cordifolia* extract; SC, *Schisandra chinensis* extract; EH, *Euphorbia humifusa* extract.

**Fig 3:**
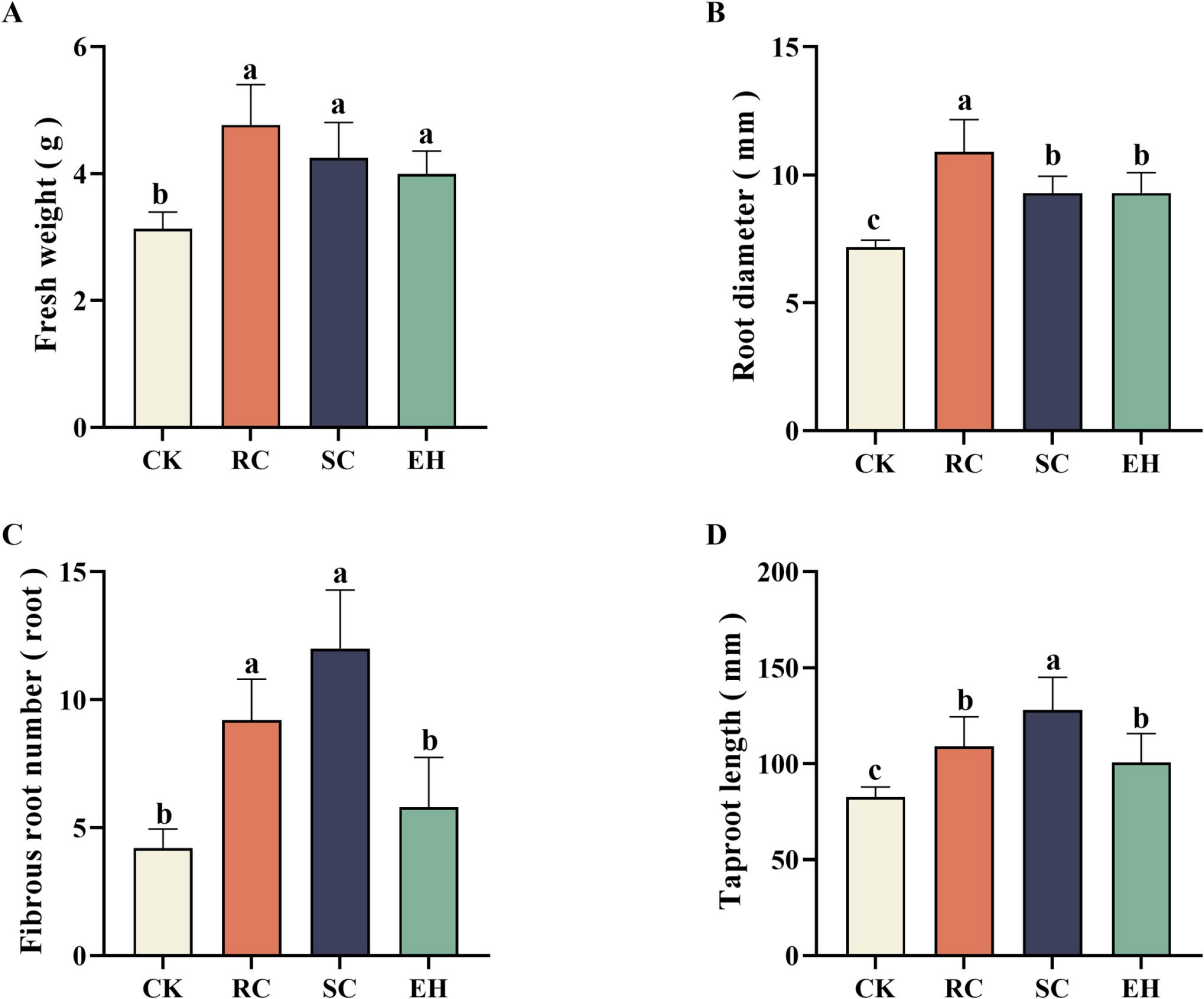
Root morphology of ginseng under different treatments. The error bars indicate the standard errors of the means of three replicates. The one-way ANOVA indicates that the letters denote a significant difference at *P* < 0.05. CK, untreated soil; RC, *Rubia cordifolia* extract; SC, *Schisandra chinensis* extract; EH, *Euphorbia humifusa* extract.

Observing the root morphology of ginseng during the harvesting period, it was found that the plant extract-related treatment was significantly better than the CK treatment, which showed that the ginseng tuber expansion was normal and disease spots were reduced (Fig 2). Meanwhile, compared to the CK treatment, ginseng fresh weight and root diameter were significantly (*P* < 0.05) increased in plant extract-related treatments, and the fresh weight and root diameter of RC treatment was the highest, increasing by 52.20% and 51.88%, respectively (Fig 3A and 3B). Compared to the CK treatment, both RC and SC treatments significantly (*P* < 0.05) increased the number of ginseng fibrous roots, while there was no significant difference between the EH treatment and CK treatment (Fig 3C). The taproot length of ginseng in all plant extract-related treatments was significantly (*P* < 0.05) higher than that in the CK treatment, and the taproot length of SC treatment was the highest, increasing by 54.86%, but there was no significant difference between RC and EH treatments (Fig 3D).

Investigation of the incidence rate and disease severity of ginseng root showed that plant extract-related treatment could reduce the incidence rate and disease severity of ginseng root (Table 2). In particular, the incidence rate and disease severity of ginseng root treated by RC were the lowest, and compared with CK treatment, the incidence rate of ginseng root treated by RC decreased by 38.89%, and the disease severity index of ginseng root decreased by 1.8.

**Table 2:** Incidence rate and disease severity of ginseng under different treatments.

Treatment	Incidence rate (%)	Disease severity
CK	83.33	4.02
RC	44.44	2.22
SC	55.56	2.78
EH	66.67	3.22

### Root activity of ginseng

**Fig 4:**
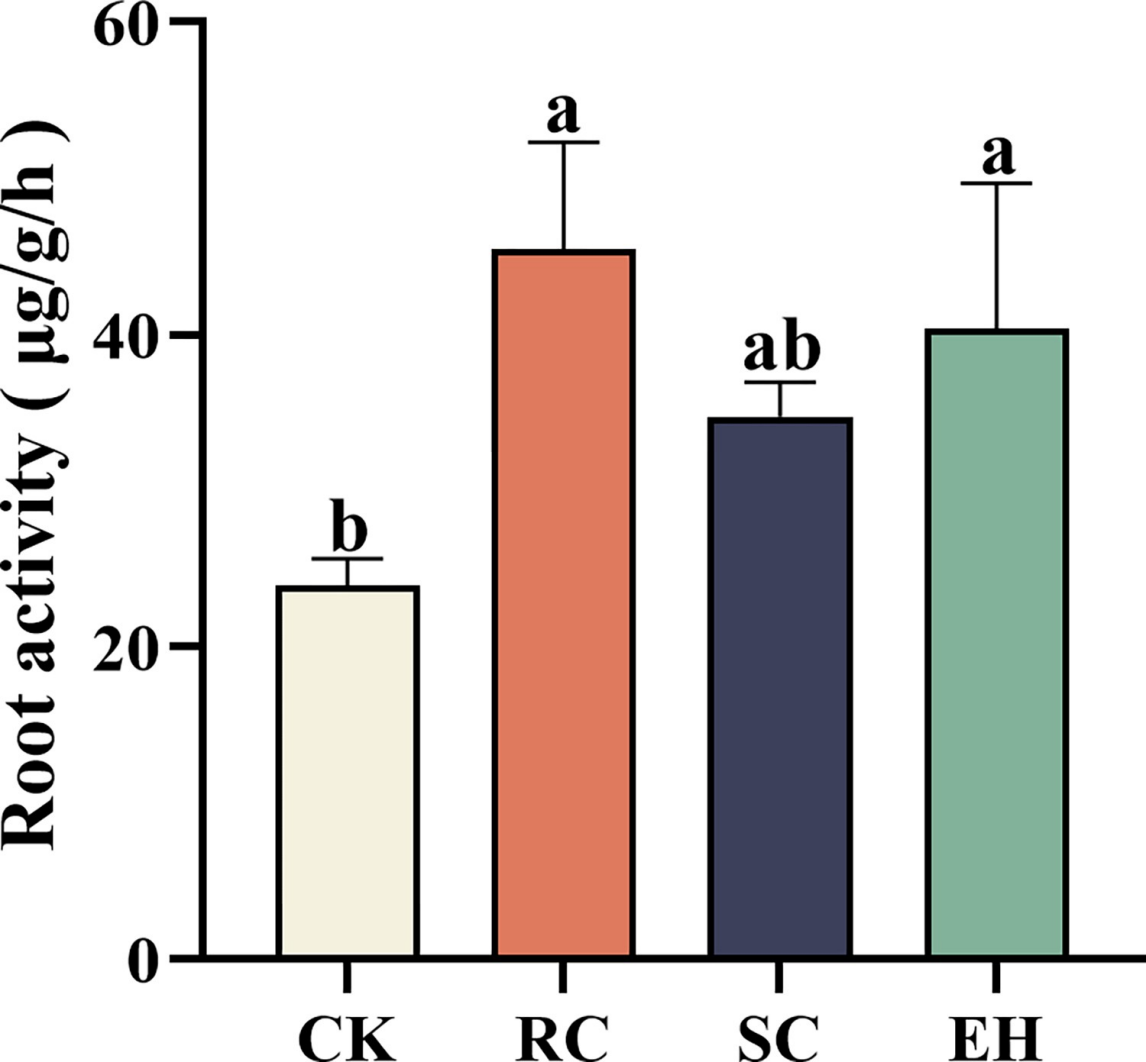
Root activity of the ginseng under different treatments. The error bars indicate the standard errors of the means of three replicates. The one-way ANOVA indicates that the letters denote a significant difference at *P* < 0.05. CK, untreated soil; RC, *Rubia cordifolia* extract; SC, *Schisandra chinensis* extract; EH, *Euphorbia humifusa* extract.

Analysis of the collected ginseng samples showed a clear difference in the root activity of ginseng treated with plant extract-related (*P* < 0.05, Fig 4). Compared with CK treatment, the ginseng root activity was significantly (*P* < 0.05) increased in RC, SC, EH treatments, and the root activity of RC treatment was the highest, whereas there were no significant differences between the RC, and EH treatments (Fig 4).

### Physiological properties of ginseng

**Fig 5:**
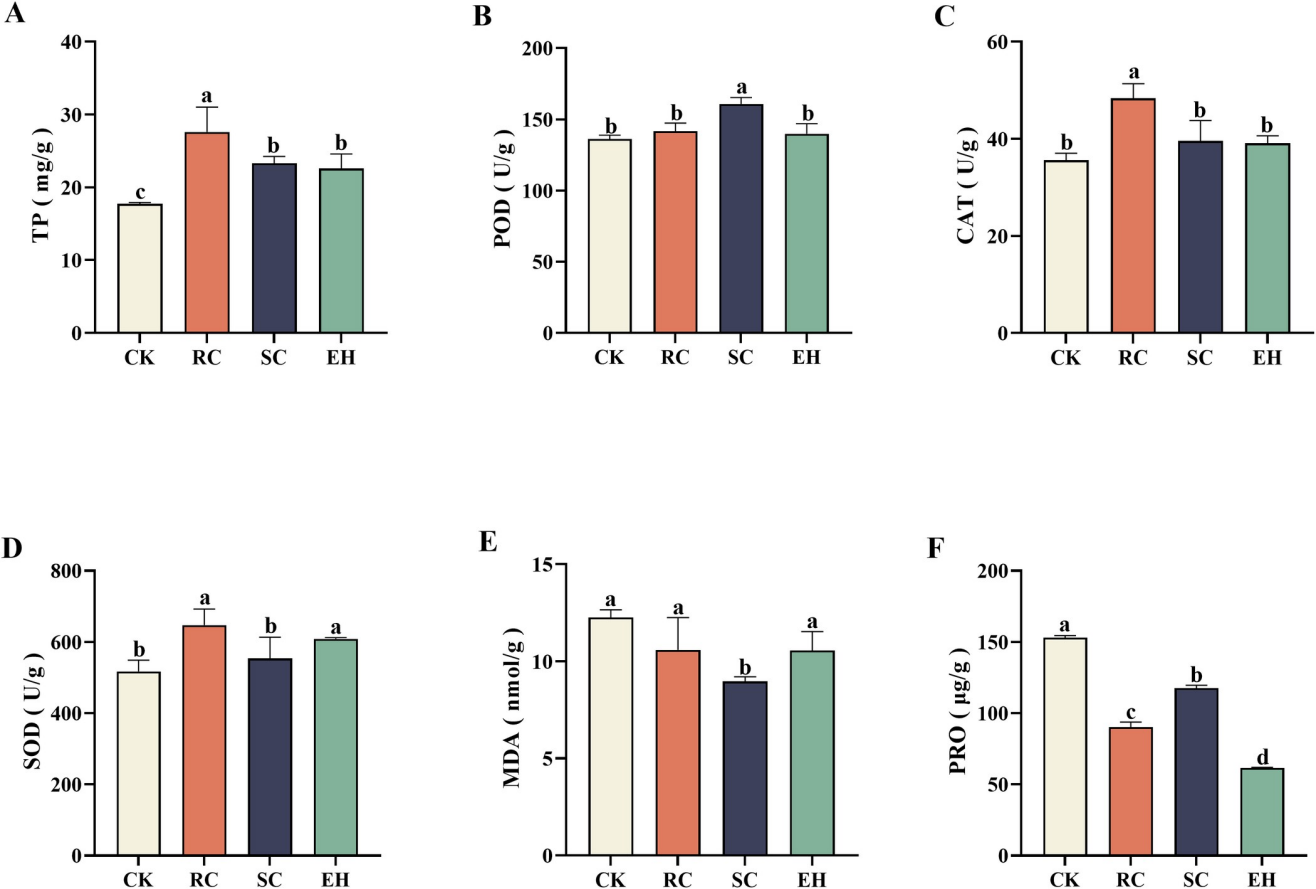
Ginseng’s physiological properties under different treatments. The error bars indicate the standard errors of the means of three replicates. The one-way ANOVA indicates that the letters denote a significant difference at *P* < 0.05. TP, Total protein; POD, peroxidase; CAT, catalase; SOD, superoxide dismutase; MDA, malondialdehyde; PRO, proline. CK, untreated soil; RC, *Rubia cordifolia* extract; SC, *Schisandra chinensis* extract; EH, *Euphorbia humifusa* extract.

Analysis of the collected ginseng samples showed a clear difference in the physiological properties of ginseng treated with plant extract-related treatments (*P* < 0.05, Fig 5). Ginseng TP content in all plant extract-related treatments was significantly (*P* < 0.05) higher than that in the CK treatment, and the ginseng TP content in the RC treatment was the highest (Fig 5A). Compared to the CK treatment, the POD activity was significantly (*P* < 0.05) increased in the SC treatment, while it was increased slightly in the RC and EH treatments, with no significant differences compared to the CK treatment (Fig 5B). Compared to the CK treatment, the CAT activity was significantly (*P* < 0.05) increased in the RC treatment, while it was increased slightly in the SC and EH treatments, with no significant differences compared to the CK treatment (Fig 5C). Ginseng SOD activity in RC and EH treatment was significantly (*P* < 0.05) higher than that in the CK treatment, while it was increased slightly in the SC treatment, with no significant differences compared to the CK treatment (Fig 5D). Ginseng MDA content was significantly (*P* < 0.05) reduced in SC treatment, while it was reduced slightly in the RC, and EH treatments, with no significant differences as compared to the CK treatment (Fig 5E). Compared to the CK treatment, the PRO content was significantly (*P* < 0.05) reduced in RC, SC, and EH treatments, and the PRO content of the EH treatment was the lowest (Fig 5F).

### Correlation analysis between fresh weight of ginseng and root system parameters

**Fig 6:**
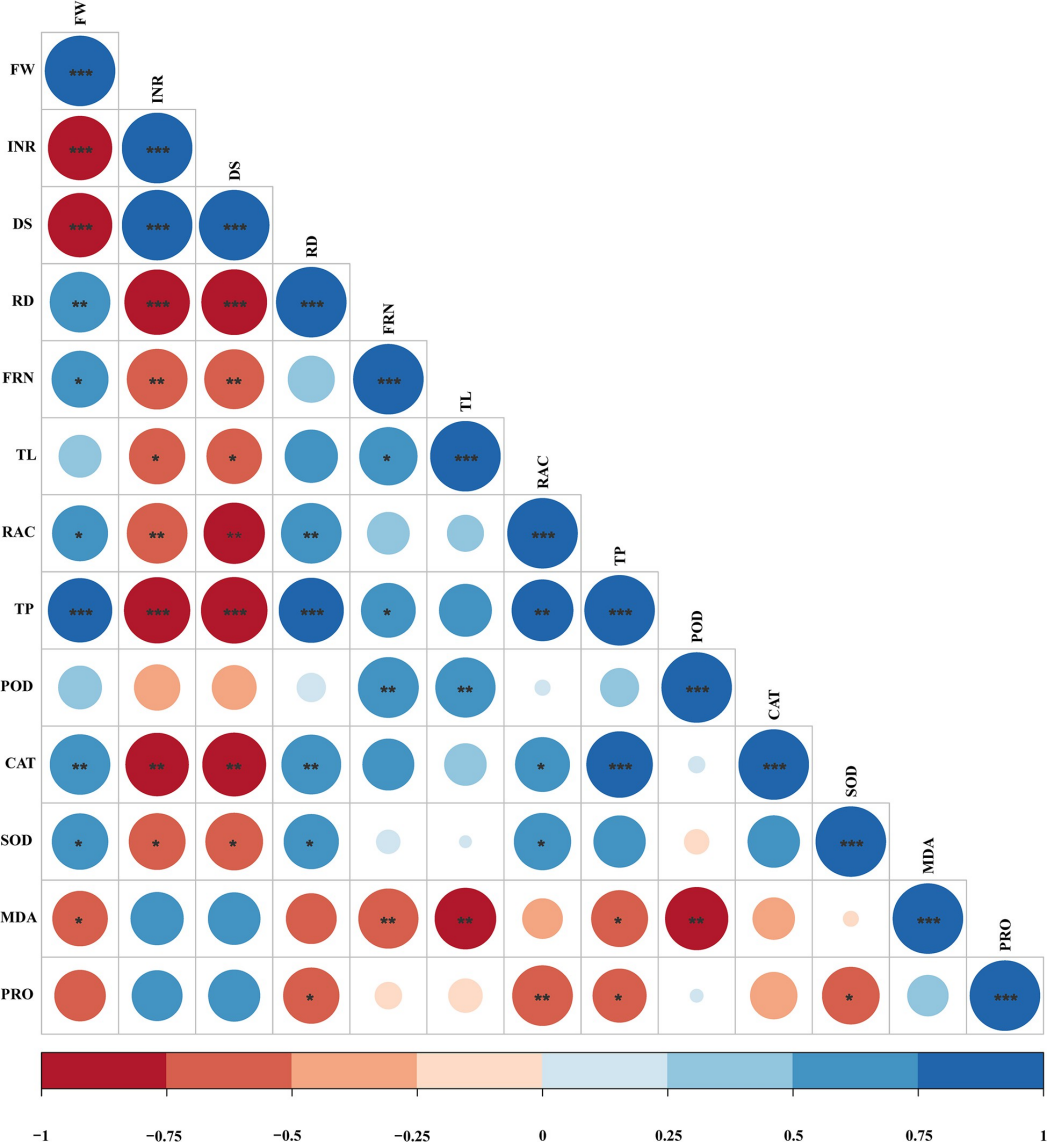
Correlation heatmap between fresh weight of ginseng and root system parameters.

Analyzed the correlation between fresh weight of ginseng and root system indicators, and drew a heatmap. As shown in Fig 6, fresh weight is significantly positively correlated with root diameter, fibrous root number, root activity, total protein (TP), catalase activity (CAT) and superoxide dismutase activity (SOD), indicating that the application of plant extracts can reduce the incidence rate of ginseng, improve the antioxidant activity of ginseng roots, and promote root growth. The fresh weight was significantly negatively correlated with incidence rate, disease severity index and malondialdehyde content (MDA), indicating that the application of plant extracts could alleviate ginseng’s environmental stress and reduce its incidence rate.

### Correlation analysis between fresh weight of ginseng and soil parameters

**Fig 7:**
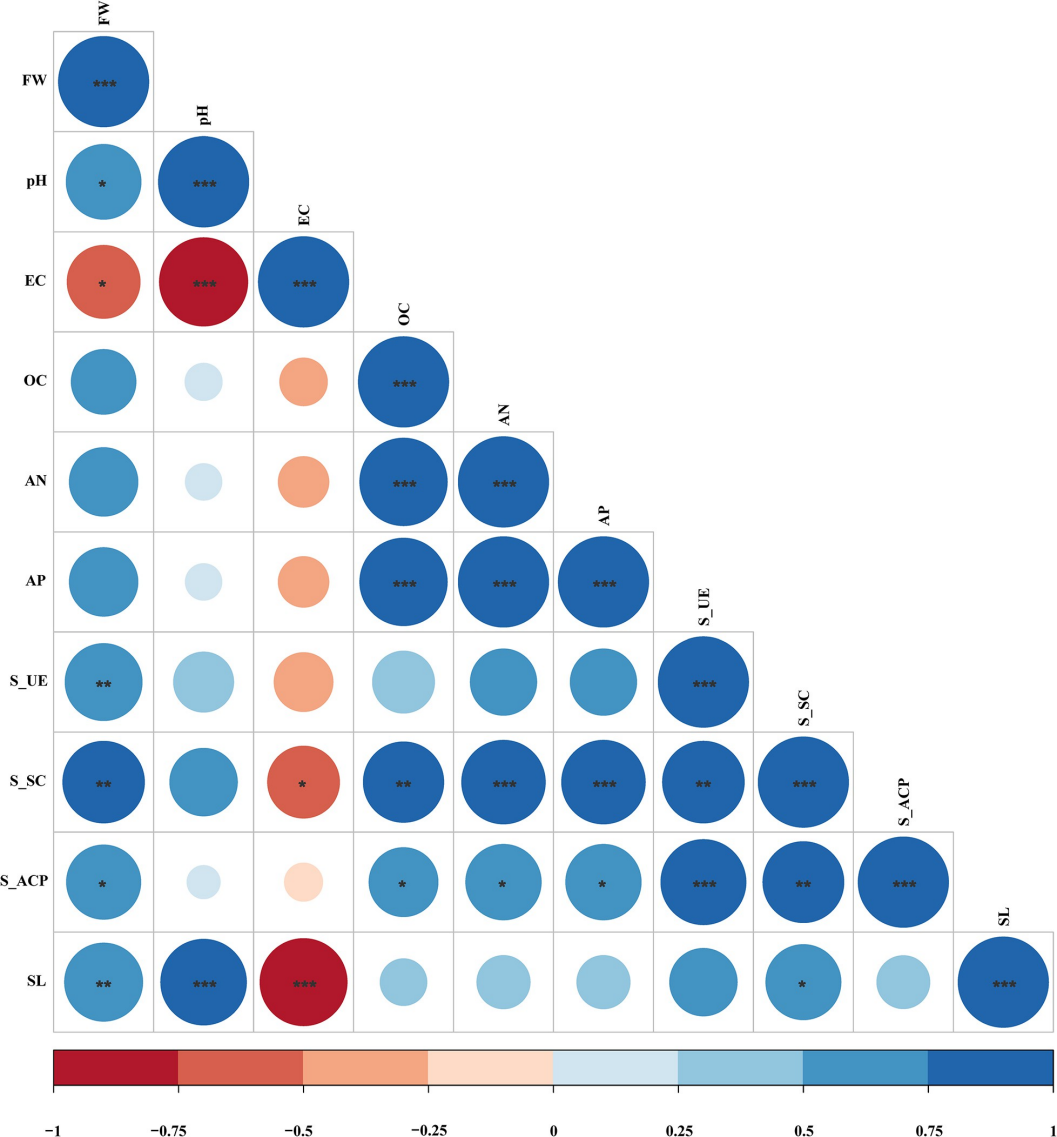
Correlation heatmap between fresh weight of ginseng and soil index.

Correlation analysis was conducted between fresh weight of ginseng and soil parameters. The result is shown in Fig 7. Fresh weight is significantly positively correlated with pH, Soil urease activity (S-UE), Soil sucrase activity (S-SC), Soil acid phosphatase activity (S-ACP), and Soil laccase activity (SL), indicating that the application of plant extracts can improve the growth of ginseng, which is related to the increase of S-UE, S-SC, S-ACP, SL, and pH.

## Discussion

The negative effects of continuous cropping are the main factor impeding the sustainable and healthy development of ginseng [38]. Developing an effective and environmentally friendly approach to overcoming these effects should therefore have high priority in sustainable cultivation of ginseng. The joint effects of different plant extracts on soil properties and enzyme activities in the rhizosphere of ginseng, as well as the growth and development of ginseng were investigated in this study. Soil acidification and salinization are the two main characteristics of soil degradation, which occurs easily, particularly in long-term single-cropping production systems due to the extensive use of fertilizers [39–41]. In the present study, except for SC treatment, the EC value, a common indicator of soil salinity, was considerably declined either in all plant extract-related treatments, with the lowest value observed in the RC treatment, which is consistent with previous reports [42]. Likewise, pH values of acidified soils were generally increased after plant extract treatment, but the degree of increase was slightly different, which was consistent with our results [16]. These changes are likely driven by specific microbial taxa, with particular functional genes, that may flourish considerably during the soil because of the plant extracts’ addition [43]. Soil organic matter is one of the important indicators for evaluating soil fertility and soil quality, which can promote plant growth and development, decompose nutrients, and improve soil properties [44]. In the present study, the OC content was significantly increased in all plant extract-related treatments, with the highest content observed in the EH treatment, which is consistent with previous reports [45]. This may be due to the small molecule of organic matter produced by plant extracts applied in the soil becoming a part of the soil organic matter. Additionally, the effects of different treatments on the large amounts of elements and their transformation in soil are not consistent in different studies [46, 47]. In the correlation heatmap (Fig 7), pH is positively correlated with fresh weight of ginseng, EC plants are negatively correlated with fresh weight of ginseng, while the correlation between OC, AN, AP and fresh weight of ginseng is not significant. We found that the contents of AP, and AN were increased in plant extract-related treatments, which may be directly due to the degradation of plant extracts, or indirectly due to the enhancement of nutrient cycling.

Soil enzymes are catalysts for soil ecosystems, playing an important role in soil material cycling, energy flow, and other aspects, that are important driving forces for soil ecological functions and system metabolism, and can be used as one of the indicators for soil quality evaluation [48, 49]. Soil urease plays a crucial role in the nitrogen cycle by catalyzing the hydrolysis of urea in the soil to produce ammonia [50]. Soil acid phosphatase plays an important role in the mineralization of soil organic phosphorus and affects the content of phosphorus compounds in the soil [51, 52]. The increase in acid phosphatase content after extract treatment facilitated the decomposition of phosphorus in ginseng-cultivated soils and significantly increased the amount of fast-acting phosphorus in the soil. Previous studies have shown that laccase promotes the formation of soil humus and organic matter [53]. In the present study, the significant increase in soil organic matter content after the treatment with *Rubia* extract may be related to the increase in laccase content. As shown in Fig 7, the fresh weight of ginseng is significantly positively correlated with S-UE, S-S, S-ACP, and SL, indicating that the extract may enrich beneficial microorganisms for ginseng by increasing soil enzyme activity, thereby promoting ginseng growth [54]. This may be because most of the enzymes in the soil are mainly secreted by soil microorganisms, however, microorganisms that act as activators of some enzymes may act as inhibitors of others [55].

Maintaining soil health is recognized as an important prerequisite for the successful alleviation of replant failure; however, the growth of multiple cropping seedlings is a key indicator for evaluating soil health [56, 57]. Our results indicated that CK treated ginseng root had more disease spots and lower fresh weight, taproot length, root diameter, fibrous root numbers, and root activity. The fresh weight of ginseng is significantly positively correlated with root diameter, fibrous root number and root activity, while the incidence rate and disease severity index of ginseng are significantly negatively correlated with fresh weight, root diameter, fibrous root number, main root length and root activity (Fig 6), indicating that the application of plant extracts can enhance the root activity and nutrient uptake rate of ginseng, thus promoting the growth of ginseng and reducing the incidence rate of ginseng. However, applying plant extract treatment, can facilitate the growth of ginseng and improve root performance, which is consistent with previous studies [58], but there are significant differences in the effects between different plant extract treatments. This was probably due to the positive effects of plant extract on chemical properties and enzymatic activity in the soil, for example, mechanisms that increase nutrient supply and inhibit soil-borne pathogens, thus reversing some of the negative effects of previous cropping systems on plant growth.

The metabolic system for scavenging reactive oxygen in the plant is unbalanced during the continuous single-planting process which leads to the accumulation of reactive oxygen species, causing damage to the plant membrane system [59, 60]. Adversity stress can affect the antioxidant enzyme system of plants, leading to the accumulation of oxygen free radicals and membrane lipid peroxidation. The product of membrane lipid peroxidation, MDA, can cause serious damage to the cell membrane system and affect plant growth [57]. Therefore, the level of MDA content is also one of the indicators reflecting the degree of stress damage to plants. MDA gradually increased, indicating that these plants were suffering from oxidative damage during this process, and SOD, POD, CAT, and PRO were all shown to respond similarly [61–63]. In our study, the MDA content of CK treatment was higher, while the MDA content of plant extract related treatment was lower, indicating that the extract can reduce membrane lipid peroxidation and reduce the degree of cell membrane damage. This may be because the increase in MDA content in untreated soil inhibits the activity of protective enzymes and exacerbates cell membrane damage [57]. Meanwhile, SOD can catalyze the disproportionation reaction in the body, converting superoxide radicals into O_2_ and H_2_O_2_, while CAT and POD clear the H_2_O_2_ produced by SOD decomposition, causing it to decompose into non-toxic H_2_O and O_2_ [64, 65]. Plant extract-related treatments all increased the CAT, SOD, and POD activities of ginseng, with RC treatment having the highest CAT and SOD activities and SC treatment having the highest POD activity. This may be because plants mainly rely on CAT and POD to jointly convert toxic H_2_O_2_ into H_2_O, resulting in a dynamic equilibrium of SOD dominated disproportionation reactions [66–69]. In addition, during the degradation process of PRO, toxic substances are produced, leading to a decrease or complete loss of enzyme activity in plants [70], which is consistent with our research results. Our study found that plant extract related treatments significantly reduced PRO content, indicating that plant extract can maintain a balance of reactive oxygen species (ROS) metabolism, maintain a balance of enzyme activity in the plant body, and protect the healthy growth of ginseng. As shown in Fig 6, the fresh weight of ginseng is significantly positively correlated with CAT and SOD, and significantly negatively correlated with MDA; The incidence rate and severity index of ginseng were negatively correlated with CAT and SOD. It may be because plant extracts enhance the physiological resistance of ginseng, reduce physiological stress, promote ginseng growth, and improve ginseng disease problems. Obviously, plant extracts have great potential in improving soil health and crop growth. Due to the diverse biological activities of many plants in the plant kingdom [71], applying their extracts to soil may affect the abundance and structure of soil microorganisms, thereby altering the physiological and biochemical indicators of the soil and affecting crops.

## Conclusion

Soil is necessary for plant growth, and soil properties and enzyme activity are critical indicators for evaluating soil. In particular, plant extract-related treatments increased soil OM, AN, AP contents, and S-UE, S-SC, and S-ACP activities. Plant extract-related treatments significantly increased plant root activity and TP content, and decreased MDA and PRO content. Furthermore, plant extract-related treatments also significantly promote fresh weight, the root length, root diameter, and fibrous root number of ginseng, and reduce the incidence rate of ginseng. However, the feedback of soil and ginseng on different extraction solutions is not the same, possibly due to the different types of bioactive compounds in different extracts, which cause different biological irritants to the soil and ginseng. More detailed research is needed to further understand the mechanism by which plant extracts improve soil health, and it is particularly important to examine the practical application of plant extracts under field conditions. In summary, suitable plant extracts have the sustainable potential to improve the quality of degraded soil and increase crop yield in soil management and crop production. This research result is beneficial for us to explore the long-term effects of extracts on soil and a wider range of crops, and promote the development of sustainable agriculture.

Highlights: 1. Plant extracts improve degraded soil quality;
2. Plant extracts promote ginseng growth;
3. Provide an agricultural measure with sustainable potential.

## Supporting information

S1 Data (XLSX)
